# Feedforward Artificial Neural Network-Based Model for Predicting the Removal of Phenolic Compounds from Water by Using Deep Eutectic Solvent-Functionalized CNTs

**DOI:** 10.3390/molecules25071511

**Published:** 2020-03-26

**Authors:** Rusul Khaleel Ibrahim, Seef Saadi Fiyadh, Mohammed Abdulhakim AlSaadi, Lai Sai Hin, Nuruol Syuhadaa Mohd, Shaliza Ibrahim, Haitham Abdulmohsin Afan, Chow Ming Fai, Ali Najah Ahmed, Ahmed Elshafie

**Affiliations:** 1 Department of Civil Engineering, Faculty of Engineering, University Malaya, Kuala Lumpur 50603, Malaysia; laish@um.edu.my (L.S.H.); n_syuhadaa@um.edu.my (N.S.M.); haitham.afan@gmail.com (H.A.A.); 2 Nanotechnology & Catalysis Research Centre, University of Malaya, Kuala Lumpur 50603, Malaysia; 3 Department of Materials Science and Metallurgy, University of Nizwa, Birkat Al Mawz 616, Oman; m.hakim@unizwa.edu.om; 4 Department of Civil Engineering, Al-Maarif University College, Ramadi 31001, Iraq; 5 Institute of Ocean and Earth Sciences (IOES), University of Malaya, Kuala Lumpur 50603, Malaysia; shaliza@um.edu.my; 6 Institute of Sustainable Energy (ISE), Universiti Tenaga Nasional (UNITEN), Selangor 43000, Malaysia; Chowmf@uniten.edu.my; 7 Institute of Energy Infrastructure (IEI), Universiti Tenaga Nasional (UNITEN), Selangor 43000, Malaysia; Mahfoodh@uniten.edu.my

**Keywords:** water quality, deep eutectic solvents, carbon nanotubes, feedforward back propagation neural network, adsorption

## Abstract

In the recent decade, deep eutectic solvents (DESs) have occupied a strategic place in green chemistry research. This paper discusses the application of DESs as functionalization agents for multi-walled carbon nanotubes (CNTs) to produce novel adsorbents for the removal of 2,4-dichlorophenol (2,4-DCP) from aqueous solution. Also, it focuses on the application of the feedforward backpropagation neural network (FBPNN) technique to predict the adsorption capacity of DES-functionalized CNTs. The optimum adsorption conditions that are required for the maximum removal of 2,4-DCP were determined by studying the impact of the operational parameters (i.e., the solution pH, adsorbent dosage, and contact time) on the adsorption capacity of the produced adsorbents. Two kinetic models were applied to describe the adsorption rate and mechanism. Based on the correlation coefficient (R^2^) value, the adsorption kinetic data were well defined by the pseudo second-order model. The precision and efficiency of the FBPNN model was approved by calculating four statistical indicators, with the smallest value of the mean square error being 5.01 × 10^−5^. Moreover, further accuracy checking was implemented through the sensitivity study of the experimental parameters. The competence of the model for prediction of 2,4-DCP removal was confirmed with an R^2^ of 0.99.

## 1. Introduction

### 1.1. Background

Phenolic compounds are easily found in industrial wastewater and they are discharged in large amounts into rivers and other natural water sources [1,2]. One common phenolic compound example is 2,4-dichlorophenol (2,4-DCP), which is recognized as one of the recalcitrant regular by-products generated from aerobic degradation of the antifungal and antibacterial agent “Triclosan (TCS, 5-chloro-2-(2,4-dichlorophenoxy)phenol)” that is usually added to healthcare products [3,4,5]. It is familiar for its strong odor, carcinogenic adverse effects, and its inability to decompose [6,7,8]. Different industries dispose of 2,4-DCP into water bodies, such as the industries of fungicides, disinfectants, insecticides, pesticides, and pharmaceuticals [9,10]. The presence of phenolic compounds in the environment, even at low concentrations, causes high toxicity and undesirable tastes and odors, thus their removal from water is considered a great preference. The world health organization (WHO) established a maximum allowable concentration of 1 µg/L of total phenolic compounds in drinking water [11]. However, phenolic compounds are well known for their high solubility and stability, which further complicates their removal from water [12,13,14,15]. Based on this, many researchers are still attempting to remove phenolic contaminants from polluted water by developing new competent cost-effective techniques. The adsorption process is the most favored, simple, and effectual method for phenolic compounds’ removal [16,17]. A variety of adsorbents have been reported in the literature, such as chitosan [18], carbon fibers [19], carbon nanotubes (CNTs) [20], activated carbons [21,22,23,24,25], and biosorbents [26].

CNTs have been used as efficient adsorbents for different types of inorganic pollutants [27,28,29,30,31] and organic pollutants [32,33,34,35,36], and they possess significant efficiency against radioactive compounds [37]. Carbon nanotubes (CNTs) have a distinctive chemical structure, large surface area, and exhibit a significant adsorption capacity and high binding affinity for a wide range of toxic pollutants [38,39,40,41,42]. However, the applications of CNTs in aqueous solutions are restricted due to their poor dispersion and agglomeration, which lead to a reduction of CNTs’ surface area, affecting their capability to remove particular compounds [43,44]. These limitations can be solved by the CNT functionalization process, which is an auspicious step to eliminate CNTs impurities, generate new functional groups, and eventually enhance CNTs performance in different fields [45,46,47].

Deep eutectic solvents (DESs) have been highlighted as a novel, prominent, and inexpensive alternative solvent for ionic liquid and other conventional chemical solvents [48,49]. DESs are composed of two or more of low-cost and green constituents, specifically salt and hydrogen bond donor (HBD). Their physiochemical properties have been widely examined and their applications in many fields have been documented, such as in chemistry, electrochemistry, biology, and more recently, in nanotechnology-related fields [50,51,52]. Furthermore, the application of DESs as low-cost functionalization agents has been lately reported for graphene and CNTs [53,54]. The potential of using DESs as functionalization agents was confirmed by the improvement of the dispersion and the adsorption capacity of CNTs without production of any unpleasant effects on the properties or structure of CNTs [48]. 

One of the most potent soft-computing techniques that has been proposed for modeling the adsorption process is the artificial neural network (ANN) technique [55]. ANN is a robust modeling tool due to its ability to recognize and reproduce non-linear relationships between variables during the training phase in different input–output patterns, thus mapping the relationship between variables and output in a qualified way [56]. Recently, different applications have implemented the ANN technique to control, filter, predict, and address many problems, such as in engineering, marketing, medicine, defense, energy, etc. [57]. Moreover, the application of ANN for the heavy metal adsorption process was recently reported [56,58,59]. Accordingly, use of the ANN tool has promising potential to model and predict the adsorption processes of different organic pollutants from water.

### 1.2. Problem Statement and Motivation

Many variables, including the pH of an aqueous solution, contact time, dosage of the adsorbent, and initial concentrations of pollutants, have an inevitable effect on the adsorption capacity, which makes the adsorption of any pollutant an intricate process that is difficult to model or predict using conventional linear methods [60]. Artificial neural network techniques are considered a competent tool that can learn and generalize the pattern of any complex and nonlinear process. Due to that, the use of ANN techniques can identify the relationship between the different variables involved in the adsorption process. Therefore, the use of ANN can successfully decrease the required time and cost for the experimental work, as well as helping in the extraction of intricate data that cannot be observed by a human or computer system. Other advantages of ANN modeling techniques are the ability to formulate the knowledge, describe the process, and extend the experimental results. Consequently, it is possible to predict the conditions and outputs required for the adsorption of phenolic pollutants onto adsorbents similar to the one examined in this study, by only using the model generated by the suggested ANNs technique while conserving cost, time, and effort.

## 2. Materials and Methods

### 2.1. Objectives

This work was a continuation of a wider research project, including preparation, functionalization, characterization, and application of DES-functionalized CNTs for the removal of 2,4-DCP. The previous work focused mainly on the production of different novel adsorbents from functionalizing CNTs with DESs. A comprehensive study was conducted to investigate the characterizations of these adsorbents and to examine their adsorption capacity for the removal of 2,4-DCP. Whereas, in the present work, the foremost aim was to examine the accuracy of the feedforward ANN-based model (FBPNN) in predicting the adsorption capacity of the most efficient adsorbent (the adsorbent with the highest adsorption capacity) by using the actual data set prepared from the experimental work in our previous study [48]. Briefly, the former work covered only the experimental part while the current paper merely concentrated on the modelling approach. The prediction competence of the FBPNN model was investigated in this work through a sensitivity study and the value of some statistical indicators.

### 2.2. Chemicals and Materials

The chemicals used in the experimental work were multiwalled carbon nanotubes (CNTs), with qualifications of D × L 6–9 nm × 5 μm, >95% (carbon), and were provided by Sigma Aldrich (Kuala lumpur, Malaysia). 2,4-dichlorophenol (2,4-DCP) from Merck (Kuala lumpur, Malaysia) was utilized as the pollutant, with a molecular weight of 163.0 g mol^−1^. Moreover, sodium hydroxide pellets, sulfuric acid H_2_SO_4_ (95%–97%), hydrochloric acid HCL (36.5%–38%), and choline chloride ChCl (≥98%) were all supplied by Sigma Aldrich, while ethylene glycol (EG) (≥98%), acetonitrile, and methanol were supplied by Merck.

### 2.3. Preparation of DES

Choline chloride salt (ChCl) was mixed with ethylene glycol (EG) as HBD at a molar ratio of [1:2] to produce [ChCl: EG] DES [48]. Both salt and HBD were mixed for 80 min at 70 °C, until the development of a consistent, clear deep eutectic solvent (DES) [61]. The synthesized DES was kept in a moisture-controlled environment in order to be used later for CNT functionalization.

### 2.4. Functionalization of CNTs

Initially, the pristine CNTs were dried at 100 °C for a whole night. The dried CNTs were refluxed with 50% H_2_SO_4_ for 1 h at 140 °C to produce H_2_SO_4_–CNTs, which were washed with distilled water using a vacuum filtration system until the washed water became neutral, with a pH value of 7. The H_2_SO_4_–CNTs were then dried under vacuum at 100 °C for 24 h. Afterward, 200 mg of H_2_SO_4_–CNTs was mixed with 7 mL of [ChCl: EG] DES at 60 °C for 3 h to produce DES–CNTs. A vacuum filtration system was used to wash the DES–CNTs and then they were dried at 100 °C for 24 h under vacuum. 

The characterization of the developed DES–CNTs adsorbent using Fourier transform infrared (FTIR), Raman spectroscopy, zeta potential, Thermogravimetric analysis (TGA), and Brunauer–Emmett–Teller (BET) was thoroughly covered in our previous work [48]. 

### 2.5. Batch Adsorption Studies

The prepared DES–CNTs were applied as a new adsorbent to remove 2,4-DCP from the water solution. The experiments were performed using different dosages of DES–CNTs (i.e., 5, 10, and 15 mg), different values of the solution pH (2, 5.14, 6, and 10), and a range of 2,4-DCP concentrations (10–80 mg/L) [48]. Next, 50 mL of stock solution was poured into a 250-mL flask, and a mechanical system was used to shake the flask at 180 rpm at room temperature. The 2,4-DCP concentration was checked at different time intervals during the adsorption process using ultra high-performance chromatography (Waters ACQUITY UPLC System, Milford, MA, USA) at a wavenumber of 285 nm. Moreover, the contact times used to determine the equilibrium time and the appropriate kinetic model were 5, 10, 20, 30, 60, 120, and 180 min and 24 h). In total, 147 samples were prepared in this study. 

### 2.6. Artificial Neural Network Model

Different types of artificial neural networks (ANNs) have been effectively applied in a wide range of fields and they have shown great performance in the fitting of non-linear functions and recognition of complicated patterns [62]. In the current study, the feed-forward back-propagation neural network (FBPNN) was applied to predict the adsorption capacity of DES-functionalized CNTs adsorbent. Generally, the structure for FBPNN consists of three main layers in a multilayer neural network: An input layer, hidden layer, and output layer. The input variables from the source are introduced in the input layer, then the hidden layer processes the signals sent by the input layer, and finally, the output layer deliver the results that have been predicted by ANN to the external receptor.

It is well known that each layer has a number of neurons and the role of each neuron is to transmit the input values and process them to the next layer. Furthermore, all layers have biases and a weight factor from the previous layer. The weight factor (W_ii_) is defined as the interaction between ANN layers and it can amend the transferred signals’ values. By adjusting the weight values of the ANN model, the optimal parameters can be selected since the FBPNN is governed by a supervisory learning algorithm technique [63]. Moreover, along with the weight factor, there are numerous FBPNN transfer functions that can modify the total information, which is, in the end, combined in the output layer [64]. The most common binary logistic sigmoid transfer function was used in this work and it can be expressed as follows:

(1) $$f(x)=\frac{1}{1+e^{-x}}$$

In the FNPNN algorithm, the input variables are forwarded into the neural network until the end of the network. Then, the output values are created and compared to the target values, and based on that the error is estimated [65,66]. Therefore, FBPNN proposes random initial weight values to find the relationship between the input data and target data, then the FBNN updates the values of weights by comparing the results between the target values and actual values. It was found that in the FBPNN model structure, there is no connection between the units of the same layer, while the weighted coefficient can express the connection between the developed layers [67]. 

### 2.7. ANN Model Development

In this work, the input variables in the FBPNN model were the concentration of 2,4-DCP, adsorbent dose, solution pH, and contact time, while the required output from the network was the adsorption capacity of DES–CNTs. The number of experimental data used for the modeling was 147, which were divided into two data sets: 122 data were utilized for the training and validation step and 25 data were utilized for the testing step. The normalization of input data was the range of (0–1) and was necessary to accelerate the back-propagation learning process [68]. The MATLAB R2014a computational platform (The MathWorks, Inc., Natick, Massachusetts, USA) was used in the current study to code and optimize the structure of the used ANN model.

**Figure 1:**
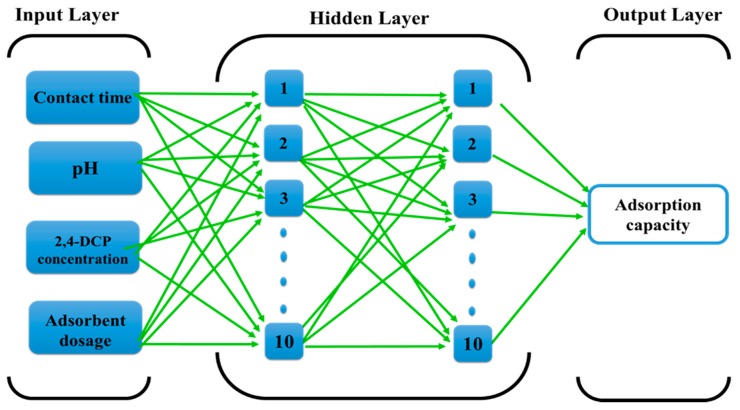
The proposed feed-forward back-propagation neural network structure.

The FBPNN model was applied for the simulation of the adsorption capacity of DES–CNTs for the removal of 2,4-DCP from water solution. Three parameters were defined as input variables, including 2,4-DCP concentration (10, 20, 30, 40, 50, 60, 70, and 80 mg/L), aqueous solution pH (2, 5.14, 6, and 10), dose of adsorbent (5, 10, and 15 mg), and finally, the contact time, which ranged from 5 min until the adsorption process reached equilibrium at 360 min. In addition, the adsorption capacity (Q) (mg/g) was defined as an output parameter. The created FBPNN model was comprised of one input layer, one output layer, and two hidden layers, with 10 neurons in each layer. The proposed architecture of the FBPNN used in this study is demonstrated in Figure 1. 

The back-propagation training function (trainbr) was applied to update the values of the weight and bias in regard to the momentum [69]. Furthermore, the training algorithm adopted for this model was the Levenberg–Marquardt (LM) training algorithm. The LM algorithm was essentially designed to overcome the limitations of the Gauss–Newton (GN) algorithm and the steepest-descent method by blending their premium attributes, and serves as a hybrid optimization algorithm [70]. The LM algorithm is more robust and has more stable convergence than thee GN algorithm; on the other hand, it is faster than the steepest-descent method, and therefore it can be considered as a bridge between the GN algorithm and the steepest-descent method. Based on that, the LM algorithm is conveniently used for a wide range of real-world applications and it is employed for training of medium- and small-sized problems in the artificial neural network field [71].

The selected function used to transfer the functions for the ANN networks was the tangent sigmoid transfer function (tansig). The stopping criterion is one of the main elements in the FBPNN model, which is developed by specific given patterns that allow the network to learn to its maximum potential by enabling it to identify its minimum acceptable error rate [72]. In this study, the stopping criterion for the FBPNN model was established based on setting up two important parameters: MSE, which was set to 0.00001 as a performance goal, and the epoch number, which was determined to be 1000. The stopping criteria are the main elements. The selection of the node number at each hidden layer was based on the training and testing of the network by using various neuron numbers upon examination of the value of the mean square error (MSE) for the testing data set.

### 2.8. Performance Indicators

The value of the mean square error (MSE) was used to calculate the error that occurs between the desired data and predicted data by the ANN model. It was administered at the stage of data training; it usually decreases at the beginning of the training stage, whereas the error, f, training begins to rise when over-fitting starts to occur. The increase of the training error, as well as the return of the minimum value of weight, results in the training stopping. The MSE value was described by the following equation:

(2) $$MSE=\frac{1}{n}{\sum_{i=1}^{n}\left(Q_{a(t)}-Q_{s(t)}\right)}^{2}$$

where $Q_{a}$ is the actual value of the adsorption capacity and $Q_{s}$ is the simulated value of the adsorption capacity.

The accuracy of the ANN model is usually evaluated by the predicted and actual results through the implementation and calculation of various indicators. The ANN model behavior can be described by different indicators, for instance, the root mean square error (RMSE), mean square error (MSE), relative error (RE), mean absolute percentage error (MAPE), and the relative root mean square error (RRMSE). The maintained indicators were defined by the following formulas:

(3) $$RRMSE={\left[\frac{1}{n}\sum_{t=1}^{n}{\left(\frac{Q_{a(t)}-Q_{s(t)}}{Q_{a(t)}}\right)}^{2}\right]}^{\frac{1}{2}}$$

(4) $$RMSE={\left[\frac{1}{n}\sum_{t=1}^{n}{\left(Q_{a(t)}-Q_{s(t)}\right)}^{2}\right]}^{\frac{1}{2}}$$

(5) $$MAPE=\frac{1}{n}\sum_{t=1}^{n}\left|\frac{\left(Q_{a(t)}-Q_{s(t)}\right)}{Q_{a(t)}}\right|\times 100$$

(6) $$RE=\frac{Q_{a(t)}-Q_{s(t)}}{Q_{a(t)}}\times 100$$

Generally, the performance of the ANN model is assessed by RRMSE, MSE, RMSE, MAPE, and RE indicators. The calculation of all indicators depends on a comparison of the estimated error of the simulated results and the actual results. The smallest the evaluated error, the better the model performance achieved.

## 3. Results and Discussion

### 3.1. Characterization of DES–CNTs and Adsorption Studies

During the experimental work [48], a complete characterization was carried out for DES–CNTs adsorbent to investigate the changes to the pristine CNTs after functionalization with [ChCl: EG] DES. It was found that the surface area of the pristine CNTs increased from 123.54 to 193.10 m^2^/g and that can be explained by the ability of DES to remove the impurities on the surface of pristine CNTs. This was confirmed by the TGA results, which revealed that the CNTs functionalized with H_2_SO_4_ and [ChCl: EG] DES had significantly high purity. Moreover, the DES–CNTs had low activation energy for oxidation because of the existence of oxygen-containing functional groups on the surface of the DES–CNTs adsorbent, which was confirmed by the FTIR results. These functional groups include hydroxyl groups (O−H) that appeared in the peaks around ~3460 cm^−1^, carboxyl groups (–COOH) at ~ 1650 cm^−1^, and carbonyl groups (C=O) at ~1400 cm^−1^. Raman spectroscopy showed that there is an obvious increase in the ration of the D band intensity (I_D_) to the G band intensity (I_G_), which is another indicator of the presence of new functional groups on DES–CNTs adsorbents. In addition, the hydrophilicity of the hydrophobicity properties of these functional groups remarkably increased the absolute value of the zeta potential for pristine CNTs from −5.5 to −24.8 mV for DES–CNTs. All of these results proved the efficiency of DES as a functionalization agent for CNTs while conserving their unique structure [48]. 

The fitting of pseudo first-order and pseudo second-order kinetic models was examined [48]. It was found that the adsorption mechanism is explained well by the pseudo second-order kinetic model, which suggests that the rate of 2,4-DCP adsorption onto DES–CNTs adsorbents is governed by chemisorption. Furthermore, the isotherm studies were also performed by applying four kinetic isotherm models. Based on the R^2^ value of each model, it was confirmed that the Langmuir isotherm model yielded the best fit, with an outstanding value of maximum adsorption capacity (Qmax) of 390.35 mg/g [48]. 

### 3.2. ANN Model Performance

**Figure 2:**
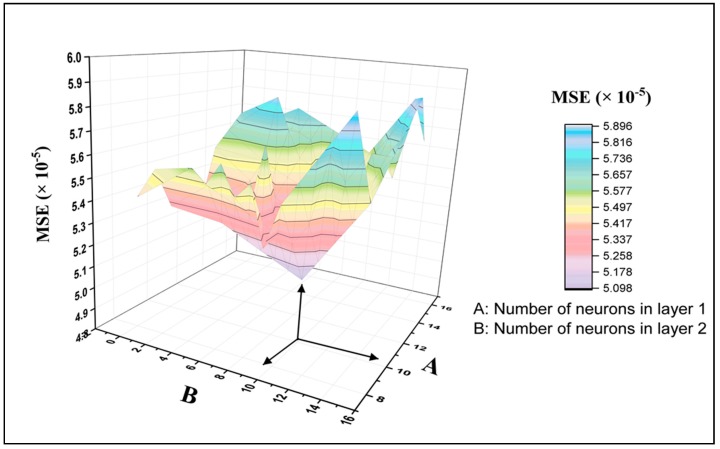
Neural networks’ performance-based MSE (the mean square error) utilizing different architectures: One and two hidden layers.

**Figure 3:**
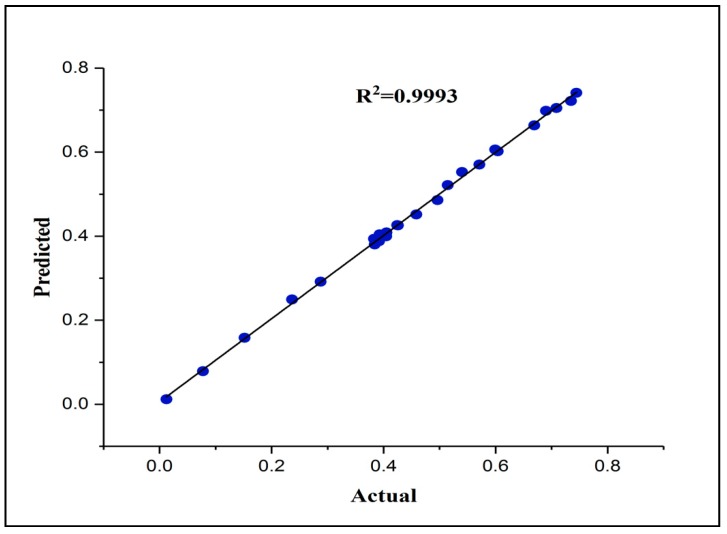
The correlation coefficient of the actual and predicted normalized adsorption capacity of DES–CNTs for 2,4-DCP removal (testing dataset).

Figure 2 illustrates the process of trial and error for selecting the neural network architecture. In this figure, the Z axis and X axis represent the number of neurons in the hidden layer one and two, respectively, while the Y-axis represents the MSE values. Once the number of neurons in the hidden layer two equals zero, then it refers to a single layer architecture. This figure clearly shows the variation in the MSE results when creating various neural network architectures. The lowest MSE presents the best architecture. It is noticeable that the architecture with 10 neurons in hidden layer one and two has the best performance compared to the other architectures, with a recorded MSE value of 5.01 × 10^−5^. Also, it can be concluded that the network with two hidden layers displayed better prediction performance than that with one hidden layer. It is worth noting from Figure 3 that the data predicted by the eFBPNN model obviously complies with the actual data acquired from the experimental work, with a correlation coefficient value (R^2^) of 0.99. 

**Figure 4:**
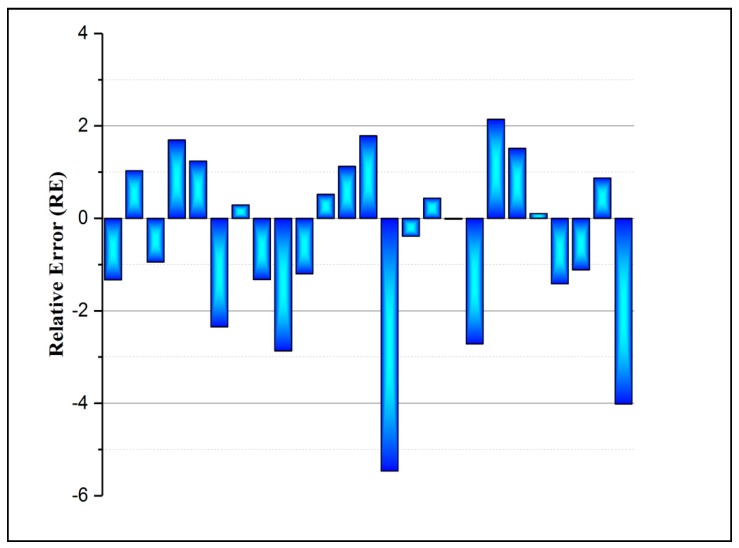
Illustration of the accuracy of the FBPNN model based on the testing dataset.

The validity of the trained FBNN model was further investigated by checking the values of some other indicators, including the root mean square error (RMSE), mean absolute percentage error (MAPE), and relative root mean square error (RRMSE). Table 1 shows the results for all the aforementioned indicators. Moreover, one of the important error indicators for the effectiveness of the model is the relative error percentage (RE) and it is depicted in Figure 4. It can be noticed that the highest RE value of the FBPNN model is 5.47%. The training of the neural network model is a substantial step in achieving good prediction performance. The key objective of this study was to obtain a benefit from the mathematical approach in real-time experiments. 

### 3.3. Sensitivity Study

#### 3.3.1. Effects of Initial Pollutant Concentration

**Figure 5:**
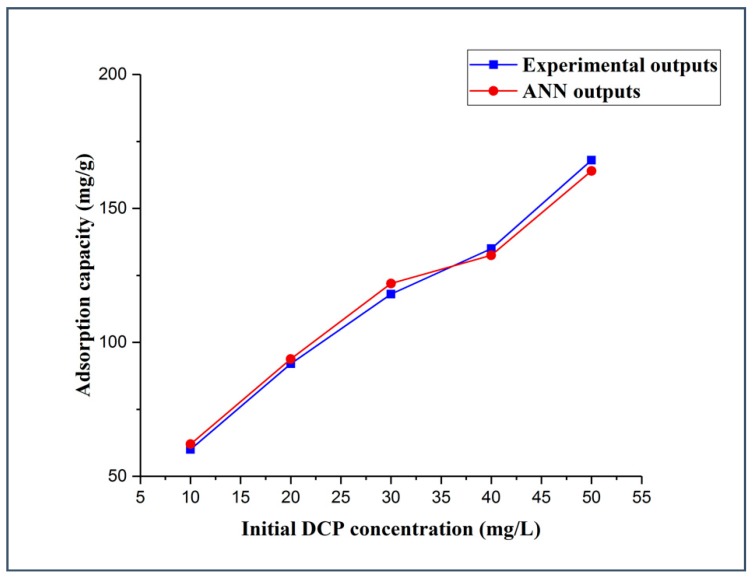
Experimental and FBPNN outputs as a function of the initial concentration.

The initial concentration effect of 2,4-DCP was explored using five different initial concentration values of 10, 20, 30, 40, and 50 mg/L, whereas all other experimental parameters were kept constant, including the adsorbent dosage (5 mg), solution pH (5.14), and contact time (120 min). It is clear from Figure 5 that by increasing the initial concentration of 2,4-DCP, the adsorption capacity of DES–CNTs increases. The adsorption capacity was increased from 60 to 92 mg/g when increasing the initial concentration of 2,4-DCP from 10 to 20 mg/L while increasing the initial 2,4-DCP concentration from 40 to 50 mg/L increased the adsorption capacity from 135 to 168 mg/g. These remarks can be explained by the fact that the driving force of the mass transfer is highly dependent on the concentration of the adsorbate; they become great at high concentrations, thus increasing the uptake capacity of 2,4-DCP from aqueous solution. However, the removal efficiency of the adsorbent was decreased when increasing the initial concentration of 2,4-DCP due to the saturation of the adsorbent active sites [73]. The actual data was used in the training of the FBPNN model technique. It is evident from Figure 5 that the observations for the FBPNN model outputs are compatible with that of the experimental outputs.

#### 3.3.2. Effect of Aqueous Solution pH

**Figure 6:**
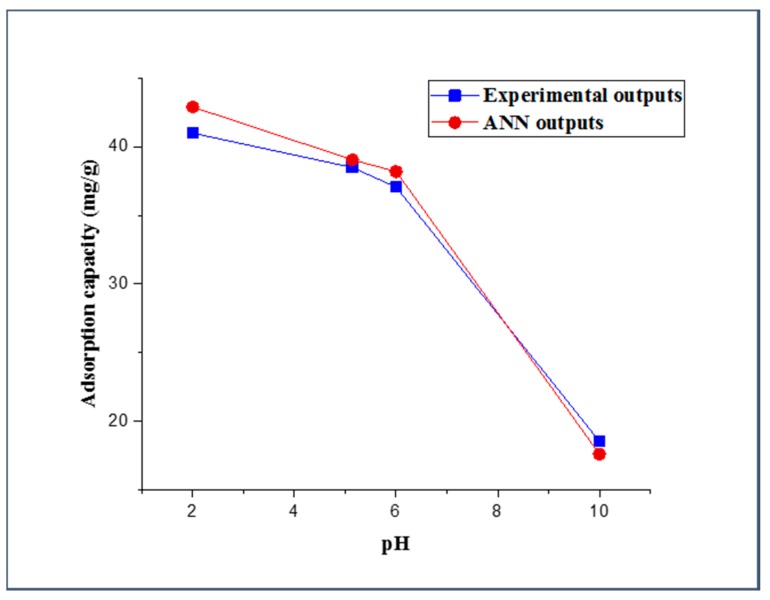
Experimental and FBPNN outputs as a function of pH.

The adsorption process is highly dependent on the value of the aqueous solution pH. The pH value has a significant effect on the protonation of the functional groups onto the adsorbent surface, such as carboxyl, phosphate, and amino functional groups, and it has been shown to display a noticeable impact on the solubility of the adsorbate [74,75]. Examination of the pH effect was performed by using three different values of aqueous solution pH (5.14, 6, and 10), and all other experimental parameters were set at constant values (i.e., adsorbent dose (5 mg), 2,4-DCP initial concentration (10 mg/L), and contact time (20 min)). The actual results and FBPNN outputs were plotted as a function of the pH and are presented in Figure 6. It can be perceived from the figure that the adsorption capacity from the experimental work decreased with the increase of the pH value. This decrease in the 2,4-DCP uptake capacity can be substantiated by the broad existence of OH^−^ in the solution deprotonating some functional groups on the adsorbent surface, which leads to more negative-charged sites [76]. Additionally, at high pH values, 2,4-DCP molecules are more likely to dissociate into the form of C_6_H_3_Cl_2_O^−^. Consequently, electrostatic repulsion will occur between the dissociated 2,4-DCP form and the negatively charged adsorption sites, resulting in a low adsorption capacity [77]. The trend of the FBPNN-predicted adsorption capacity as a function of the pH value accurately concurs with the trend of the experimental results.

#### 3.3.3. Effects of Adsorbent Dosage

**Figure 7:**
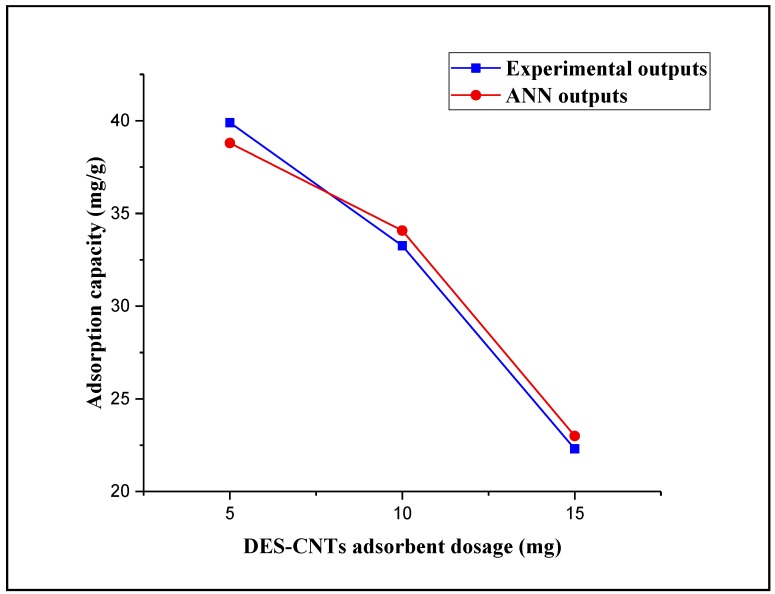
Experimental and FBPNN outputs as a function of the adsorbent dosage.

Three different dosages of DES–CNTs adsorbent (5, 10, and 15 mg) were used in this study to examine their effect on the process of 2,4-DCP adsorption. This experiment was carried out at a constant contact time of 20 min, a constant pH value of 2, and an initial concentration of 2,4-DCP of 10 mg/L. Figure 7 shows the trend of adsorbent dosage versus the experimental outputs and ANN outputs. It is apparent that the adsorption capacity of DES–CNTs adsorbent decreased with the increase of its dosage in the polluted water. The recorded adsorption capacity for the 15-mg dosage of DES–CNTs adsorbent was 22.3 mg/g and it was increased to 33.26 and 39.9 mg/g as the adsorbent dosage decreased to 10 and 5 mg, respectively. The possible justification for this observation is that as the dosage of the adsorbent increases, the surface of the adsorbent will increase and more active sites will be presented. Consequently, the 2,4-DCP uptake capacity will decrease [60,78]. The FBPNN model technique was used to predict the effect of the adsorbent dosage by using the data obtained from the experiments in training and prediction. Figure 7 presents the predicted data from FBPNN and the experimental outputs as a function of the adsorbent dosage. It can be concluded that the observation of the FBPNN outputs agrees with that of the experimental outputs.

#### 3.3.4. Adsorption Kinetics Study

**Figure 8:**
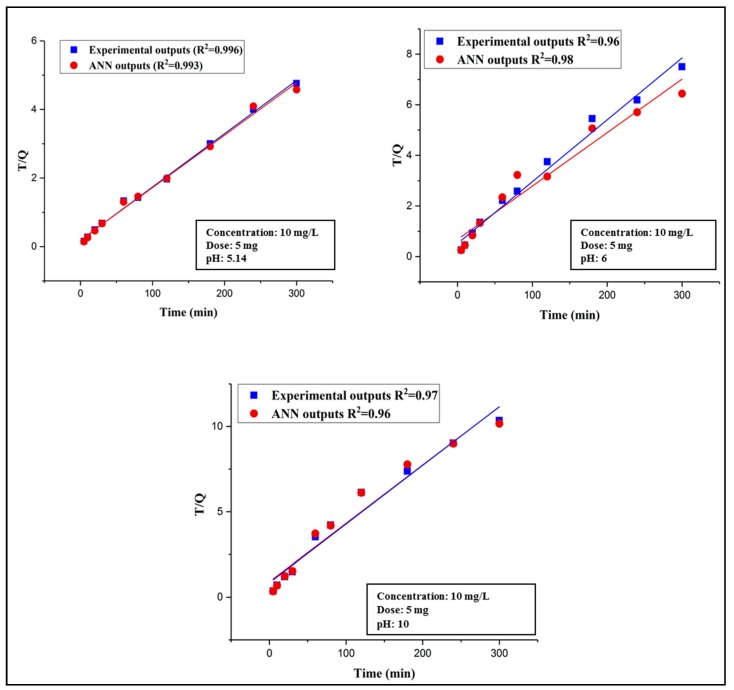
Kinetics study (Pseudo second-order model) at different pH values.

The kinetic study was mainly carried out to examine the DCP removal and adsorption rate and its adsorption mechanism onto DES–CNTs. Two well-known kinetic models were applied to both the experimental outputs and FFBP neural network outputs, i.e., pseudo first-order and pseudo second-order. The kinetic study was investigated by using a constant adsorbent dosage of 5 mg and by varying the pH values (5.14, 6, and 10) with an initial 2,4-DCP concentration of 10 mg/L. The validity of each kinetic model was confirmed by the value of the correlation coefficient (R^2^) for both the predicted and experimental data. The best kinetic model that explained the adsorption of 2,4-DCP was the pseudo second-order model and it is illustrated in Figure 8 at different pH values for the ANN outputs and actual outputs. The correlation coefficients of all studied kinetic models are listed in Table 2. The results from the kinetic studies indicate that the adsorption mechanism involves both the DES–CNTs adsorbent and the adsorbate and suggests that chemisorption controls the rate of the adsorption process [48]. A similar manner was reported for various types of adsorbents in previous studies [79,80]. 

The data that were acquired from the experimental work were modeled and predicted using the FBPNN technique. The kinetic study was also performed on FBPNN outputs by applying the same three kinetic models. By checking the correlation coefficient (R^2^), it was found that the pseudo second-order model was more adequate in describing the kinetics of this adsorption study compared to the pseudo first-order and intraparticle diffusion models. The R^2^ values for the kinetic study on FBPNN outputs are listed in Table 2. It is clear that the FBPNN model shows high accuracy since its results are sufficiently close to the results obtained from the experimental work.

## 4. Conclusions

The ChCl-based DES was effectively used to functionalize CNTs while conserving their important features and with no damage caused to their structure. The new adsorbent (DES–CNTs) successfully adsorbed 2,4-DCP from the water, with a maximum adsorption capacity of 390.35 mg/g. The adsorption capacity of DES–CNTs was governed by several operational parameters. The FBPNN model was sufficiently capable of predicting the adsorption of 2,4-DCP from water and that was assured by comparing the experimental outputs with the FBPNN model outputs. The minimum MSE value was 5.01 × 10^−5^ with an R^2^ value of (0.9993), which depicts good agreement between the actual data and predicted data. The accuracy of the FBPNN model was also approved by other indicators, for instance, the RMSE (7.08 × 10^−3^), RRMSE (1.94 × 10^−2^), and MAPE (1.52). It is worth mentioning that the FBPNN is theoretically considered the most common modeling technique in comparison to the other methods. Despite some of the common reported limitations and shortcomings, including a slow learning speed, local minima, and difficulty capturing the high complexity, non-stationarity, dynamism, and nonlinearity of time series, the performance of this method for the prediction of 2,4-DCP adsorption on DES–CNTs was reliably satisfactory and it can be easily applied for the prediction of the desorption process of these adsorbents, as long as no hysteresis occurred. 

## Figures and Tables

**Table 1:** Evaluation indicators.

Indicator	FBPNN
MSE	5.01 ×10^−5^
RMSE	7.08 ×10^−3^
RRMSE	1.94 ×10^−2^
MAPE	1.52

Note: MAPE = mean absolute percentage error; MSE = mean square error; RMSE = root mean square error; RRMSE = relative root mean square error.

**Table 2:** Adsorption kinetics constants and correlation coefficient for each model.

		Pseudo First Order		Pseudo Second Order		Intraparticle	
pH	C_0_ mg/L	Experimental R^2^	ANN Output R^2^	Experimental R^2^	ANN Output R^2^	Experimental R^2^	ANN Output R^2^
5.14	10	0.772	0.764	0.996	0.993	0.879	0.881
6	10	0.883	0.879	0.96	0.98	0.887	0.876
10	10	0.88	0.87	0.97	0.96	0.892	0.887
